# Impact of COVID-19 on palliative care of cancer patients: Perspectives from Pakistan

**DOI:** 10.1016/j.amsu.2022.103705

**Published:** 2022-05-13

**Authors:** Sajeel Saeed, Kashif Tousif, Chaudhary Abdul Fatir, Jawad Basit, Ka Yiu Lee, Muhammad Junaid Tahir

**Affiliations:** a Holy Family Hospital, Rawalpindi Medical University, Rawalpindi, Pakistan; b Lahore General Hospital, Lahore, 54000, Pakistan; c Swedish Winter Sports Research Centre, Department of Health Sciences, Mid Sweden University, Östersund, Sweden

**Keywords:** Cancer patients, Palliative care, Pandemic, Coronavirus

## Abstract

The COVID-19 pandemic has adversely affected the survival rate and palliative care of cancer patients all over the globe. In Pakistan, there are only a few institutions and organizations which provide specialized facilities for palliative care. During the pandemic, these specialized facilities were further limited. As only less than one percent of people had access to palliative care across Pakistan in the pandemic, the situation can be improved by establishing more such departments, providing telemedicine, increasing social media campaigns, and highlighting the importance of palliative care among cancer patients.

## 1 Main text

The department of palliative care is responsible to provide specialized medical care tending to relieve the pain and suffering of patients having a particular cataclysmic disease regardless of the stage of illness. According to American Society of Oncology, within eight weeks of receiving a diagnosis, every patient with advanced cancer should see a palliative care team [1]. Just like many other disciplines, the palliative care department is in great ordeal because of the coronavirus disease 2019 (COVID-19) pandemic. COVID-19 tends to have catastrophic outcomes in certain individuals especially with certain co-morbidities. In this context, cancer patients are more susceptible to life-threatening infection by this novel severe acute respiratory syndrome coronavirus 2 (SARS-CoV-2).

The mortality rate of COVID-19 among cancer patients is 25.6% [2]. Social distancing protocols had hampered the delivery of routine health care resulting in shutting down the hospitals all across the globe. To counter COVID-19, national health services of the United Kingdom had also shut all its care facilities, leading to the delay in the screening of cancer at a vast scale, and subsequently an increase in death rate ranged from 4% to 8% for lung cancer and 16.6% for colorectal cancer [3,4]. Due to high death rate and susceptibility, the European Society of Medical Oncology (ESMO) had to issue a missive to take extra care of COVID-19 infected cancer patients [5]. According to ESMO, cancer care during COVID-19 has four pillars consisting of physical, psychological, social, and spiritual care [6].

Every year, between 170,000 and 200,000 new cancer cases are estimated in Pakistan [7]. Even in the best of circumstances, cancer treatment is complicated, costly, time-consuming, and distressing for patients and their families. The COVID-19 problem has further complicated things by causing economic instability, travel restrictions, and a reduction in regular clinical activities in hospitals. This is because of the large number of patients requiring hospitalization and, surprisingly, emergency unit treatment, medical services experts are regularly redeployed from their unique specialization to COVID-19 therapy [1]. Currently, there is not a single domain for palliative care in Pakistan, only pain management services are available at Agha Khan Teaching Hospital in Karachi and Shaukat Khanam Hospital in Lahore. A survey investigating palliative care facilities around the globe conducted by the International Observatory at the End of Life Care (IOELC) has observed a “least favorable ratio” of patients receiving care in the palliative care facilities in Pakistan, as only one such service got identified for a population of more than 157 million [8]. Before the pandemic, palliative care in Pakistan included emotional support from family, delivery of a pain-relieving medication at the patient's residence, or spiritual assistance from religious experts. After the pandemic, standard operating measures for social separation were recommended, despite major religious gatherings, including regular communal prayers, remained unrestricted. Over the period, educational institutions, offices, and a large number of enterprises had been shuttered multiple times. However, officials have been hesitant to enforce a total lockdown due to concern about economic disruption. The coronavirus pandemic has had a significant influence on the therapy options available for cancer patients. Büntzel et al. stated that, according to health care workers, more than 70% of cancer patients felt insecure, with up to 21% feeling terrified and alone during the pandemic [9].

According to a report, no new cancer patients were admitted for treatment by the end of March 2020, the number of new patients starting chemotherapy had dropped by two-thirds, and the number of patients starting radiation treatment had halved [7]. Delays in accepting new patients and treating existing patients are anticipated to have a significant negative impact on the cancer survival and cure rates, both nationally and worldwide. 10.13039/100014337Furthermore, travel restrictions, lack of moral support, financial issues, and medicine shortage were additional obstacles to cancer patients’ palliative care foist by the lockdown.

Though Pakistan does have National Cancer Control Plan (for palliative treatment mainly), its enactment has not been fully carried out as less than 1% of the total population have access to palliative care facilities [10]. Before the pandemic, Pakistan's gross domestic product (GDP) for healthcare was 3.2%, which was well below the current health expenditure of our neighboring countries, for example, China (5.35%), India (3.54%), Afghanistan (9.39%) and Iran (8.66%) [11]. Furthermore, the availability of opioids, especially morphine is also sporadic. There is a need to develop effective interventions for both COVID-19-infected cancer patients and those who are vulnerable. These obstacles can be dealt with by establishing palliative care centers across the country, minimizing face-to-face contact either by telemedicine services or by online video consultations with the doctors. Telehealth has played a major role in patient care services during the pandemic. It allowed continued doctor and patient care with minimum face-to-face interaction and has become an essential strategy for providing early palliative care in outpatient settings [12].

For those who require some kind of test, the testing centers should be relocated to some local areas to avoid hospital visits. Medications may also be provided to the patients by delivery or a drive-through facility may be arranged. The healthcare budget must be increased to duly deal with untoward circumstances. A German research project has developed the “PallPan” paradigm for palliative care of patients at any stage of sickness. This project aims to address the negative experience of patients who had palliative care during the lockdown, to ensure door-to-door availability of volunteer palliative care services, to increase hospital staff dedicated to palliative care, to address the issue of end-of-life care at the national and state levels, and to increase social media campaigns and publications on the care of seriously ill patients with advanced disease [13]. Patients who are very sick or dying, as well as their families, have an added burden during the current COVID-19 pandemic. Their care is made even more complicated by protection, strict isolation measures, and visitation restrictions. Front-line practitioners should be taught to identify palliative care candidates and provide evidence-based palliative care recommendations, and facilitate reasonable resource use while preserving patient dignity and psychological health throughout the pandemic. There is a dire need to develop policies that incorporate cancer and palliative care into current healthcare systems, especially in low-and middle-income countries (LMICs), in an attempt to improve healthcare access and early diagnosis.

## Financial disclosure

None to declare.

## Ethical approval

This short communication does not require ethical approval.

## Sources of funding

None to declare.

## Author contribution

S.S and K.T conceived the idea, S.S, K.T, C.A.F, J.B and M.J.T retrieved the data and draft the manuscript, K.Y.L and M.J.T reviewed critically and provided inputs. All authors approved the final version of the manuscript.

## Trail registry number

1. Name of the registry: NOT APPLICABLE
2. Unique Identifying number or registration ID:
3. Hyperlink to your specific registration (must be publicly accessible and will be checked):

## Guarantor

Ka Yiu Lee.

Swedish Winter Sports Research Centre, Department of Health Sciences, Mid Sweden University, Östersund, Sweden.

kyle.lee@miun.se.

## Consent

Studies on patients or volunteers require ethics committee approval and fully informed written consent which should be documented in the paper.

Authors must obtain written and signed consent to publish a case report from the patient (or, where applicable, the patient's guardian or next of kin) prior to submission. We ask Authors to confirm as part of the submission process that such consent has been obtained, and the manuscript must include a statement to this effect in a consent section at the end of the manuscript, as follows: “Written informed consent was obtained from the patient for publication of this case report and accompanying images. A copy of the written consent is available for review by the Editor-in-Chief of this journal on request”.

Patients have a right to privacy. Patients’ and volunteers' names, initials, or hospital numbers should not be used. Images of patients or volunteers should not be used unless the information is essential for scientific purposes and explicit permission has been given as part of the consent. If such consent is made subject to any conditions, the Editor in Chiefmust be made aware of all such conditions.

Even where consent has been given, identifying details should be omitted if they are not essential. If identifying characteristics are altered to protect anonymity, such as in genetic pedigrees, authors should provide assurance that alterations do not distort scientific meaning and editors should so note.

Not applicable.

## Declaration of competing interest

None to declare.
